# Supplementary material for: Delineating the heterogeneity of senescence-induced-functional alterations in hepatocytes
Source: Cell Mol Life Sci. 2024 Apr 30;81(1):200. doi: 10.1007/s00018-024-05230-2 (PMC11058795; doi:10.1007/s00018-024-05230-2)
Supplement: Supplementary file 1 — Supplementary file1 (PDF 1877 KB) [file 18_2024_5230_MOESM1_ESM.pdf]

Supplementary information

Delineating the heterogeneity of senescence inducers in primary hepatocytes

Pavitra Kumar[1], Mohsin Hassan[1], Frank Tacke[1], Cornelius Engelmann[1,2*]

[1]Department of Hepatology and Gastroenterology, Charité—Universitätsmedizin Berlin, 13353 Berlin, Germany

[2] Berlin Institute of Health (BIH), 10178 Berlin, Germany

*Author to whom correspondence should be addressed.

S1. Optimization of dosage of senescence inducers by SA-β-Gal activity assay in mouse primary hepatocytes

To assess the effects of various senescence inducers on SA-β-Gal activity in mouse primary hepatocytes (MPH), we first optimized the diseases and time of treatment. We categorized the inducers based on their mode of action and observed their effects on SA-β-Gal activity.

Oxidative Stress Inducer: Hydrogen peroxide treatment at concentrations of 50 μM and 200 μM for 12 to 72 hours resulted in a significant increase in SA-β-Gal activity, indicating the induction of senescence. However, treatment with a higher concentration of 500 μM exhibited cytotoxic effects, leading to the absence of detectable SA-β-Gal activity.

DNA-Damaging Agents: Doxorubicin treatment at concentrations of 0.1 μM, 1 μM, and 5 μM for up to 48 hours increased SA-β-Gal activity in a time-dependent manner, suggesting the activation of senescence pathways. However, prolonged treatment for 72 hours reduced SA-β-Gal activity, possibly due to cytotoxicity. Etoposide, a topoisomerase II inhibitor, showed dose- and time-dependent increases in SA-β-Gal activity at concentrations of 1 μM, 10 μM, and 50 μM, suggesting the activation of senescence pathways associated with DNA damage. Cisplatin, a platinum-based chemotherapeutic agent, induced a time-dependent increase in SA-β-Gal activity at concentrations of 1 μg/ml and 5 μg/ml. Conversely, treatment with 20 μg/ml exhibited cytotoxic effects, resulting in lower SA-β-Gal activity.

Metabolic Stress Inducers: Hydroxyurea treatment at concentrations of 10 μM, 100 μM, and 500 μM increased SA-β-Gal activity in a time-dependent manner, indicating the induction of senescence through replication stress. Galactosamine treatment at a concentration of 0.1 mM showed a time-dependent increase in SA-β-Gal activity, indicating the induction of senescence. Interestingly, treatment with higher concentrations of 1 mM and 10 mM initially led to increased SA-β-Gal activity for up to 24 hours, followed by cytotoxic effects and a lower level of SA-β-Gal activity.

Epigenetic Modulator: 5-Azacitidine treatment at concentrations of 1 μM, 5 μM, and 20 μM resulted in a relatively lower increase in SA-β-Gal activity compared to other inducers. However, prolonged treatment for 72 hours with any concentration exhibited cytotoxic effects, suggesting a potential role in both senescence induction and cytotoxicity.

Telomerase Inhibitor: BIBR-1532 treatment at concentrations of 5 mM and 20 mM showed a time-dependent increase in SA-β-Gal activity, indicating the induction of senescence by telomerase inhibition. Notably, treatment with 100 mM exhibited cytotoxic effects and lower SA-β-Gal activity.

p53 Activator: Nutlin 3a treatment at concentrations of 1 μM and 10 μM induced a time-dependent increase in SA-β-Gal activity, suggesting the activation of senescence pathways

mediated by p53. However, treatment with 50 mM showed cytotoxicity effects and a lower level of SA-β-Gal activity.

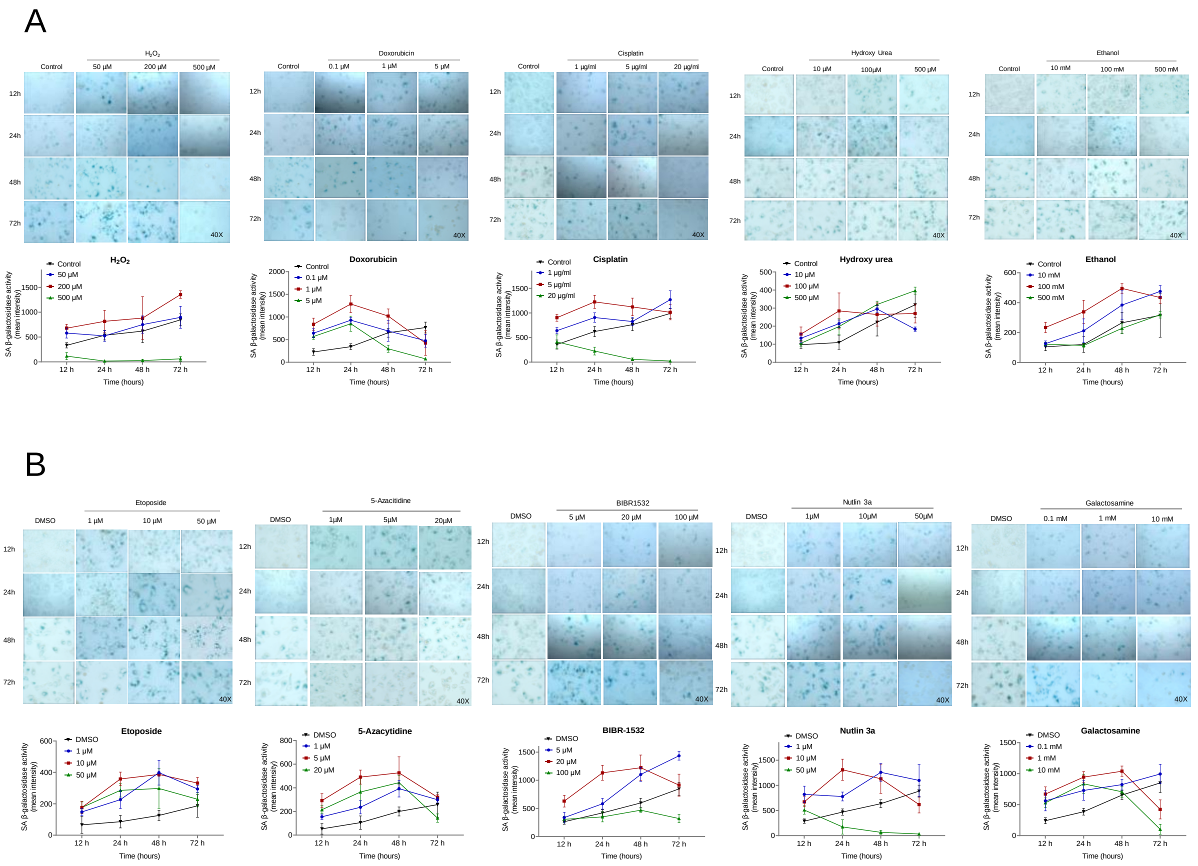

Figure S1. Senescence inducers increased senescence-associated β-galactosidase activity in mouse primary hepatocytes

Treatment time (12h, 24h, 48h, and 72h) and dosage (three doses for each drug) of drugs were optimized and the representative images are shown. The intensity was quantified with Image J. Data represent three independent experiments. A) drugs dissolved in water and B) drugs dissolved in DMSO.

S2. Assessment of cell death markers under senescence inducers in mouse primary hepatocytes

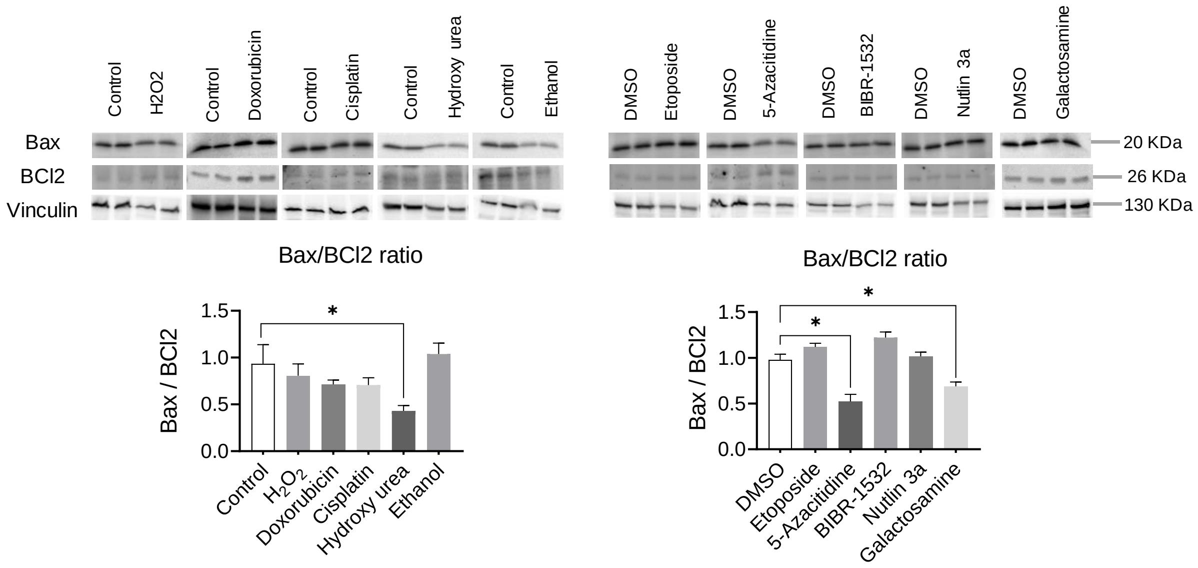

Figure S2. Effect of senescence inducers on apoptotic markers in mouse primary hepatocytes

*Representative Immunoblotting images of the effect of senescence inducers on cell cycle and proliferation markers in mouse primary hepatocytes treated for 24 hours in 2D culture. Vinculin served as a loading control. A) Drugs soluble in water and, B) relative quantification. C) Drugs soluble in DMSO and, D) relative quantification. Data is collected from four biological replicates (n=3). The band intensity was quantified using Image J. The final concentration of 200 µM H_2O_2, 1 µM doxorubicin, 5 µg/ml cisplatin, 100 µM hydroxy urea, 100 mM ethanol, 10 µM etoposide, 5 µM 5-azacitidine, 20 µM BIBR-1532, 10 µM nutlin 3a, and 1 mM galactosamine were used. Student's t-test was used to compare the effect of treatment with the respective control. Error is shown as standard deviation. (*P < 0.05, **P < 0.001, and ***P < 0.001).*

S3. Detailed images of senescence markers in mouse primary hepatocytes by immunofluorescence assessment.

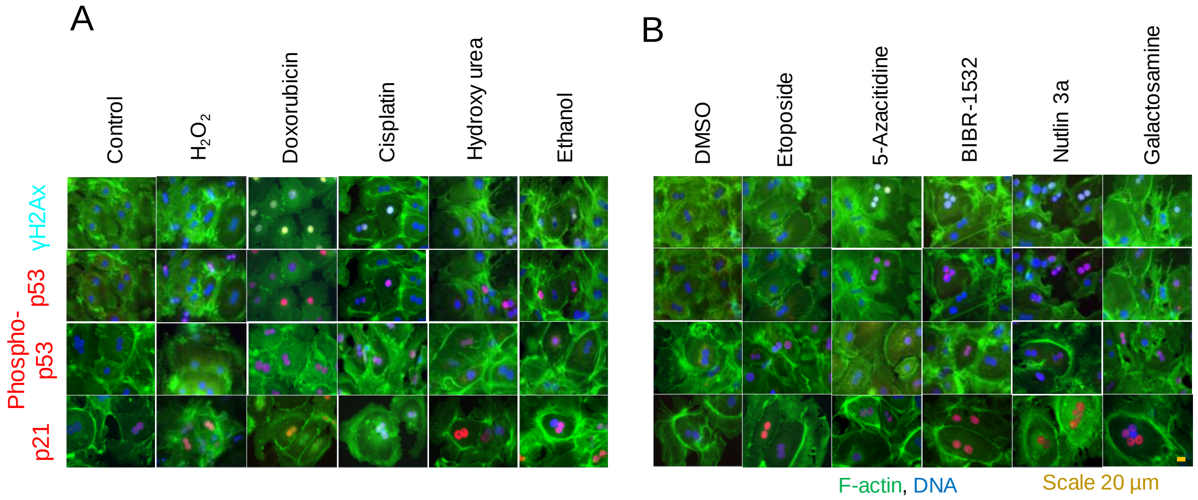

Figure S3. DNA damage response is activated differently among senescence inducers: *Immunofluorescence images represent senescence-induced DNA damage response markers in mouse primary hepatocytes treated for 24 hours in 2D culture A) Drugs soluble in water and B) Drugs soluble in DMSO. The final concentration of 200 µM* H_2O_2*, 1 µM doxorubicin, 5 µg/ml cisplatin, 100 µM hydroxy urea, 100 mM ethanol, 10 µM etoposide, 5 µM 5-azacitidine, 20 µM BIBR-1532, 10 µM nutlin 3a, and 1 mM galactosamine were used. Data is collected from four biological replicates (n=4) and five technical replicates.*

S4. Effect of senescence inducers on nuclear membrane integrity in mouse primary hepatocytes.

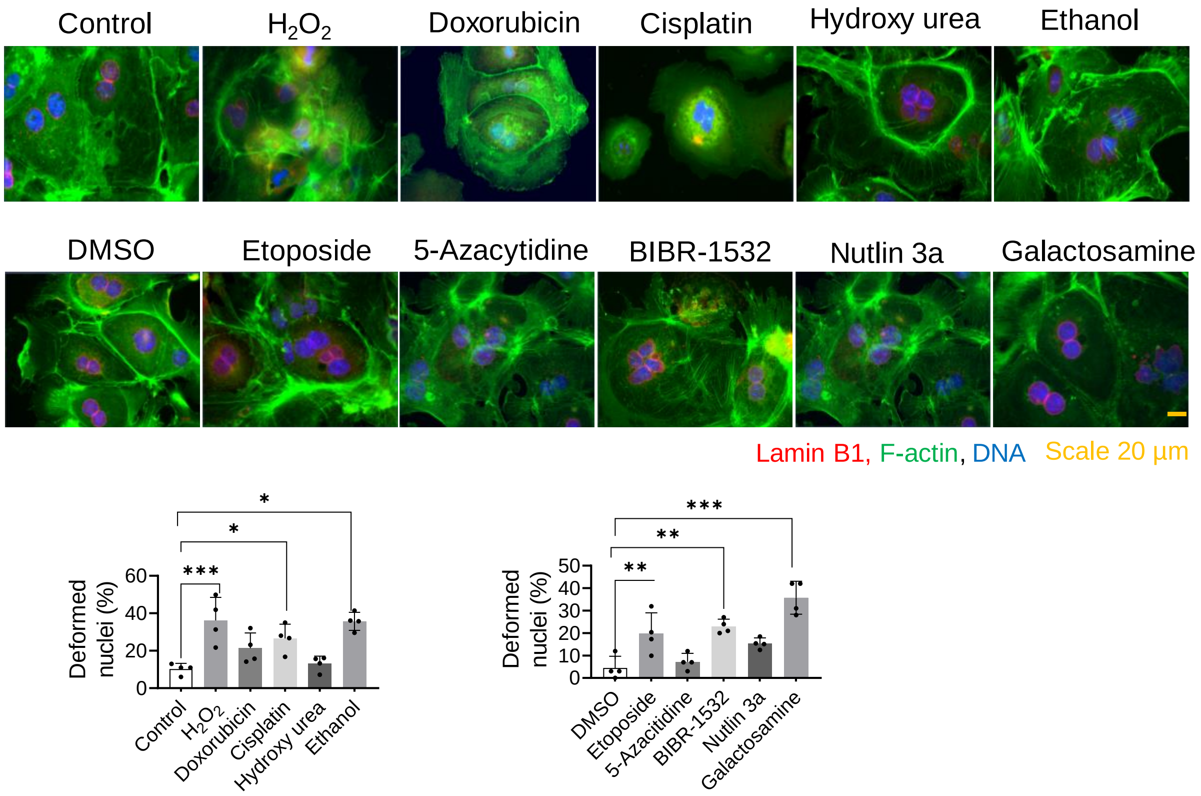

Figure S4. Nuclear membrane integrity is lost in senescent mouse primary hepatocytes

*Immunofluorescence images represent the nuclear membrane marker, lamin B1 in mouse primary hepatocytes treated for 24 hours in 2D culture. Bar graphs represent the percentage of deformed nuclei per field area. Data is collected from four biological replicates (n=4) and five technical replicates. The mean of technical replicates was used for statistical analysis. The final concentration of 200 µM H_2O_2, 1 µM doxorubicin, 5 µg/ml cisplatin, 100 µM hydroxy urea, 100 mM ethanol, 10 µM etoposide, 5 µM 5-azacitidine, 20 µM BIBR-1532, 10 µM nutlin 3a, and 1 mM galactosamine were used. Student's t-test was used to compare the effect of treatment with the respective control. Error is shown as standard deviation. (*P < 0.05, **P < 0.001, ***P < 0.001, and ****P < 0.001).*

S5. Effect of senescence inducers on gene expression of the SASP candidates

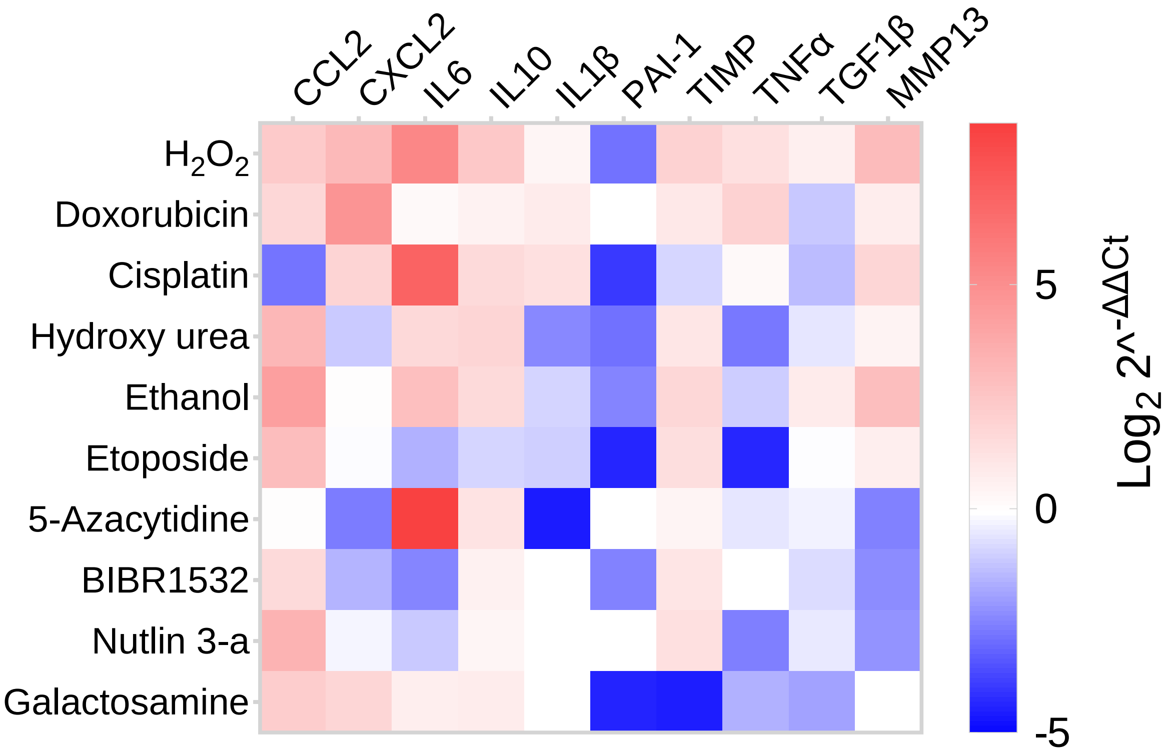

Figure S5. *Heat map representing the gene-secreted level expression of cytokines, chemokines, and matrix metalloproteases selected among the SASP candidates. GAPDH served as a reference gene and the data show the fold change over the control or DMSO group, as per the vehicle used for the respective drug. The final concentration of 200 µM H_2O_2, 1 µM doxorubicin, 5 µg/ml cisplatin, 100 µM hydroxy urea, 100 mM ethanol, 10 µM etoposide, 5 µM 5-azacitidine, 20 µM BIBR-1532, 10 µM nutlin 3a, and 1 mM galactosamine were used. The data represent the mean of three biological and two technical replicates.*

S6. Dynamic effect of senescence inducers on cellular bioenergetics

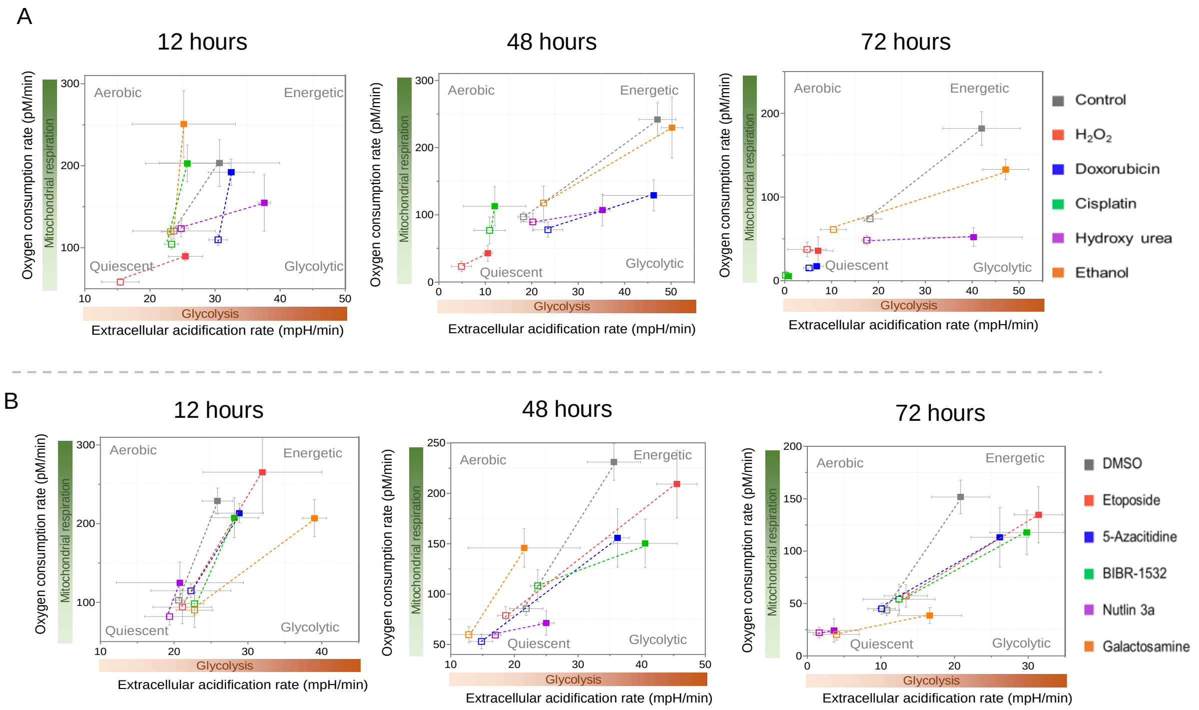

Figure S6. Oxidative phosphorylation and glycolytic capacities *measured by Seahorse flux analyzer. Basal and stressed levels were measured with 10 mM glucose, 2 mM pyruvate, and 1 mM glutamine as substrates. The final concentration of 200 µM* H_2O_2*, 1 µM doxorubicin, 5 µg/ml cisplatin, 100 µM hydroxy urea, 100 mM ethanol, 10 µM etoposide, 5 µM 5-azacitidine, 20 µM BIBR-1532, 10 µM nutlin 3a, and 1 mM galactosamine were used. Error is shown as standard deviation.*

S7. Detailed images of DNA damage response markers of senescence inducers treated hepatocyte-derived organoids.

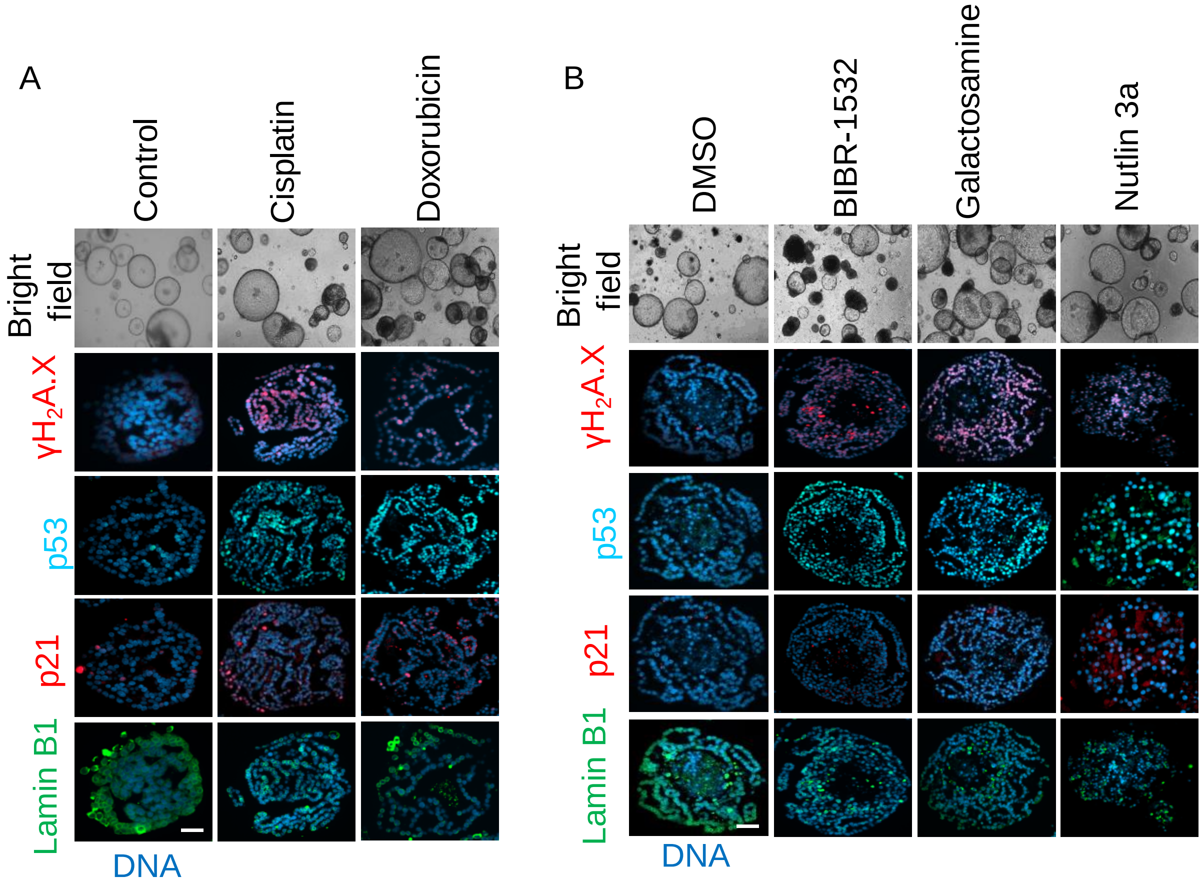

Figure S7. DNA damage response is activated differently among senescence inducers: *Immunofluorescence images represent senescence-induced DNA damage response markers hepatocyte-derived organoids. The final concentration of 200 μM H_2O_2, 1 μM doxorubicin, 5 μg/ml cisplatin, 100 μM hydroxy urea, 100 mM ethanol, 10 μM etoposide, 5 μM 5-azacitidine, 20 μM BIBR-1532, 10 μM nutlin 3a, and 1 mM galactosamine were used. The organoids were treated for 48 hours.*

Table S1. Senescence and apoptosis score of senescence inducers

S.No.	Methods	Markers	H2O2	Doxorubicin	Cisplatin	Hydroxy Urea	Ethanol	Etoposide	5-Azacytidine	BIBR-1532	Nutlin 3a	Galactosamine
1	SA β-galactosidase activity		1	1	1	0	0	0	1	1	1	1
2	Immunoflorescence	γH2A.X	0	1	1	0	1	0	1	1	1	1
3		p53	1	1	1	1	1	0	1	1	1	1
4		p_p53	0	1	1	1	1	1	0	0	1	1
5		p21	1	1	1	1	1	1	1	1	1	1
6		Deformed nuclei	1	1	1	0	1	1	0	1	0	1
7	Immunoblotting	Cyclin A2 expression	-1	1	1	0	0	1	0	0	1	1
8		CDC2 phosphorylation	-1	0	1	0	0	-1	0	0	1	1
9		CDC2 expression	1	1	1	-1	0	1	1	0	0	1
10		Histone 3 expression	1	1	1	1	1	0	0	0	0	1
11		Lamin B1 expression	0	1	1	0	-1	0	0	0	0	1
12		PCNA expression	1	1	1	0	-1	0	0	0	0	1
13	qPCR (SASP)	CCL2	1	1	-1	1	1	1	0	1	1	1
14		CXCL2	1	1	1	-1	0	0	-1	-1	0	1
15		IL6	1	0	1	1	1	-1	1	-1	-1	1
16		IL10	1	1	1	1	1	-1	1	1	1	1
17		IL1β	0	1	1	-1	-1	-1	-1	0	0	0
18		PAI-1	-1	0	-1	-1	-1	-1	0	-1	0	-1
19		TIMP	1	1	-1	1	1	1	1	1	1	-1
20		TNFα	1	1	1	-1	-1	-1	-1	0	-1	-1
21		TGF1β	1	-1	-1	-1	1	0	-1	-1	-1	-1
22		MMP13	1	1	1	1	1	1	-1	-1	-1	0
23	Immunoblotting	AKT phosphorylation	-1	-1	-1	-1	-1	0	-1	-1	0	0
24		AKT expression	0	0	1	1	1	0	1	0	0	0
25		MAPK phosphorylation	0	0	0	0	0	-1	0	-1	-1	-1
26		MAPK expression	0	1	0	0	-1	1	0	0	0	0
27		mTOR phosphorylatior	1	0	0	1	1	1	0	0	-1	1
28		mTOR expression	-1	1	-1	-1	-1	1	-1	0	1	0
29	Cellular bioenergetics	Basal ECAR	-1	0	-1	0	-1	-1	-1	-1	-1	-1
30		Stressed ECAR	-1	1	-1	1	-1	1	1	1	-1	-1
31		Basal OCR	-1	-1	-1	0	-1	0	-1	0	-1	-1
32		Stressed OCR	-1	-1	-1	-1	-1	-1	-1	-1	-1	-1
	**Over all senescence score**		**7**	**17**	**9**	**3**	**2**	**3**	**0**	**0**	**1**	**8**
	**Cell death score (Bax/BCl2 based)**		**0**	**0**	**0**	**-1**	**0**	**1**	**-1**	**1**	**0**	**-1**

Table S2. Percentage change with respect to control											
		Water as a solvent					DMSO as a solvent				
S.No.	Markers	H_2O_2	Doxorubicin	Cisplatin	Hydroxy Urea	Ethanol	Etoposide	5-Azacitidine	BIBR-1532	Nutlin 3a	Galactosamine
1	$\gamma H_2A.X$	-100.0	18150.0	2942.0	160.0	1840.0	159.3	1711.1	1411.1	2059.3	744.4
2	p53	1491.4	5257.1	1678.6	508.6	750.0	52.8	643.1	1187.5	1329.2	444.4
3	Phospho (S15)_p53	-100.0	1442.0	1050.3	-8.3	86.5	2866.7	-86.5	102.1	368.8	782.3
4	p21	209.7	212.5	100.0	284.7	179.2	379.6	471.4	591.8	626.5	379.6
5	Lamin B1	253.2	107.8	159.5	27.3	248.3	341.7	61.1	411.1	241.7	694.4
6	CCL2	419.9	242.1	-83.6	815.2	1863.8	678.4	6.2	212.4	1019.7	384.7
7	CXCL2	823.5	2667.1	279.7	-52.0	5.8	-7.6	-83.2	-65.5	-14.2	242.1
8	IL6	4200.0	14.6	8547.7	1237.2	626.6	18.3	36841.6	-81.1	1.9	46.6
9	IL10	880.2	91.0	213.9	319.0	239.1	-7.7	196.4	89.7	35.5	67.0
10	IL1β	134.5	167.3	136.3	-69.3	11.3	-37.9	-90.4	-95.6	-96.2	-96.3
11	PAI-1	-85.2	-99.3	-93.4	-86.0	-81.7	-94.7	-97.2	-81.9	-97.5	-95.0
12	TIMP	303.8	98.6	-41.7	115.0	242.8	175.5	38.0	119.5	164.5	-95.4
13	TNFα	168.0	332.7	18.7	-79.8	-47.2	-90.2	-32.7	-91.2	-83.3	-60.3
14	TGF1β	69.7	-52.5	-57.8	-29.5	81.3	-2.3	-16.8	-38.3	-26.1	-72.1
15	MMP13	1914.6	499.2	350.6	67.1	1392.6	72.6	-68.5	-66.8	-57.7	-96.3
16	SA β-gal	233.4	334.1	351.2	25.2	31.7	84.4	136.3	565.5	564.8	431.2
17	Basal ECAR	-75.3	2.5	-30.1	1.8	-14.7	-12.1	-14.2	-9.2	-39.6	-26.3
18	Stressed ECAR	-80.1	-16.5	-19.2	-4.0	-12.0	12.0	-0.2	4.5	-26.4	-10.6
19	Basal OCR	-77.9	-27.5	-32.6	-0.8	-11.8	0.1	-16.4	-4.5	-46.6	-27.9
20	Stressed OCR	-68.3	-49.4	-18.7	-50.2	-15.4	-24.0	-39.0	-32.3	-59.4	-24.7
21	AKT phosphorylation	-47.8	-69.1	-72.6	-64.3	-53.0	40.7	-63.1	-27.7	19.1	-4.5
22	AKT expression	11.7	16.7	82.5	53.2	114.0	-18.2	65.2	1.7	12.6	12.1
23	MAPK phosphorylation	29.4	-22.2	-0.6	-15.7	-11.5	-64.3	-2.4	-57.3	-68.0	-21.2
24	MAPK expression	-18.0	13.7	-5.0	2.0	-12.1	16.6	-14.5	2.6	-1.6	5.3
25	mTOR phosphorylation	57.7	-7.3	64.1	141.7	70.5	20.2	23.8	-4.3	-13.7	80.0
26	mTOR expression	-35.8	145.7	-29.3	-69.8	-65.7	57.9	-34.8	-1.5	84.5	-21.1
27	AMPK phosphorylation	46.2	3.2	17.8	16.5	32.4	-27.0	64.8	2.9	-15.5	-18.3
28	AMPK expression	-22.5	-0.2	23.0	11.3	-29.2	20.6	-17.4	-11.1	8.4	18.8
29	p62 phosphorylation	-2.0	201.0	1.4	-23.5	6.0	-14.6	6.7	5.4	28.1	22.0
30	p62 expression	-24.8	-76.8	-19.3	2.2	-17.5	13.7	8.5	-14.1	26.8	-41.7
31	CDC2 phosphorylation	276.2	93.1	-24.6	63.8	61.5	65.2	14.8	32.2	-35.7	-18.3
32	CDC2 expression	-73.2	-97.7	-78.1	2.7	-19.3	-47.4	-35.2	-4.2	-23.3	-56.1
33	Bax/Bcl2	-46.7	-52.4	-52.9	-71.3	-30.9	11.8	-47.7	22.1	1.6	-31.2
34	Cyclin A2 expression	74.2	-92.0	-56.7	25.0	-1.7	-35.6	23.7	14.4	-88.1	-67.8
35	Histone 3 expression	-31.3	-72.1	-72.1	-44.2	-68.4	-27.1	-27.1	-8.5	-16.8	-39.8
36	Lamin B1 expression	-2.9	-57.1	-57.1	30.4	70.0	11.9	11.9	-21.1	10.6	-45.7
37	PCNA expression	-33.5	-45.3	-25.8	40.3	79.9	24.6	-28.5	7.3	-32.6	-63.6
38	VDAC1	-13.9	-20.4	-2.9	20.9	23.3	-8.5	50.9	-25.7	10.5	-7.7
39	COX IV	32.3	-12.7	21.0	-31.9	-27.3	10.8	8.8	24.5	31.2	3.3

Table S3. Detailed information for antibodies used

Primary antibodies

S.No.	Antibody	Company	Cat. Number	Dilution
1	ɣH2A.X	Abcam	ab81299	1:400 for IF
2	p53	Cell Signaling Technology	2524	1:200 for IF, 1:1000 for WB
3	Phospho (S15) p53	Cell Signaling Technology	9284	1:200 for IF
4	p21	Abcam	ab188224	1:400 for IF, 1:1000 for WB
5	Lamin B1	Proteintech	66095-1-Ig	1:300 for IF, 1:5000 for WB
6	VDAC1	Proteintech	10866-1-AP	1:100 for IF
7	Cyclin A2	Abcam	ab181591	1:2000 for WB
8	Phospho CDC2	Cell Signaling Technology	9111	1:1000 for WB
9	CDC2	Cell Signaling Technology	28439	1:1000 for WB
10	Histone 3	Cell Signaling Technology	4499	1:2000 for WB
11	PCNA	Abcam	Ab29	1:1000 for WB
12	Vinculin	Merck millipore	CP74	1:1000 for WB
13	Phospho AKT	Cell Signaling Technology	9271	1:1000 for WB
14	AKT	Cell Signaling Technology	9272	1:1000 for WB
15	Phospho MAPK	Cell Signaling Technology	4631	1:1000 for WB
16	MAPK	Cell Signaling Technology	9212	1:1000 for WB
17	Phospho mTOR	Cell Signaling Technology	5536	1:1000 for WB
18	mTOR	Cell Signaling Technology	2972	1:1000 for WB
19	VDAC1	Abcam	ab14734	1:1000 for WB
20	COX IV	Proteintech	11242-1-AP	1:5000 for WB

Secondary antibodies

S.No.	Antibody	Company	Cat. Number	Dilution
1	Goat anti-rabbit	BioRad	1721019	1:10,000 for WB
2	Goat anti-mouse	BioRad	1721011	1:10,000 for WB
3	Anti-rabbit AF-647	Cell Signaling Technology	4414	1:500 for IF
4	Anti-rabbit AF-555	Cell Signaling Technology	4413	1:500 for IF
5	Anti-mouse AF-647	Cell Signaling Technology	4410	1:500 for IF
6	Anti-mouse AF-555	Cell Signaling Technology	4409	1:500 for IF

Table S4. Primers for qPCR

		Primers for qPCR	
**S.No.**	**Gene**	**Forward primer sequence (5'-3')**	**Reverse primer sequence (5'-3')**
**1**	CCL2	GTGTTGGCTCAGCCAGATGC	GACACCTGCTGCTGGTGATCC
**2**	CXCL2	CCCAGACAGAAGTCATAGCCAC	TGGTTCTTCCGTTGAGGGAC
**3**	IL6	GCTACCAAACTGGATATAATCAGGA	CCAGGTAGCTATGGTACTCCAGAA
**4**	IL10	GGCTGAGGCGCTGTCATCG	TCATTCATGGCCTTGTAGACACC
**5**	IL1β	GAGCTGAAAGCTCTCCACCTC	CTTTCCTTTGAGGCCCAAGGC
**6**	PAI-1	GACACCCTCAGCATGTTCATC	AGGGTTGCACTAAACATGTCAG
**7**	TIMP	CCAGAACCGCAGTGAAGAGT	GTACGCCAGGGAACCAAGAA
**8**	TNFα	ACCACGCTCTTCTGTCTACTGA	TCCACTTGGTGGTTTGCTACG
**9**	TGF1β	ATTCAGCGCTCACTGCTCTT	ATGTCATGGATGGTGCCCAG
**10**	MMP13	CCTTCTGGTCTTCTGGCACAC	GGCTGGGTCGTCACACTTCTCTGG
**11**	MMP2	CAACGGTCGGGAATACAGCAG	CCAGGAAAGTGAAGGGGAAGA
**12**	HPRT1	CAAACTTTGCTTTCCCTGGT	TCTGGCCTGTATCCAACACTTC
**13**	GAPDH	TGTTGAAGTCACAGGAGACAACCT	AACCTGCCAAGTATGATGACATCA
